# Supplementary material for: Are carbon emissions trading and green financial instruments synergistic? -Comprehensive quantitative research based on content analysis
Source: PLoS One. 2024 Mar 7;19(3):e0298601. doi: 10.1371/journal.pone.0298601 (PMC10919601; doi:10.1371/journal.pone.0298601)
Supplement: S1 Data — (ZIP) [file pone.0298601.s001.zip › Data/地级市Z_F工作报告.zip!/║╙─╧/Σ≡║╙╩╨/└┤╫╘╣1⁄2╓┌║┼í╛┬φ┐╦╩2╛▌═°í┐_2010.docx]

各位代表:

我代表市人民政府，向大会作工作报告，请予审议，并请各位政协委员提出意见。

折叠2009年工作回顾

2009年，是新世纪以来我市经济发展最为困难的一年。面对国际金融危机的严重冲击，在市委的正确领导下，在市人大、市政协的监督支持下，市政府团结带领全市人民，迎难而上，共克时艰，较好地完成了市五届人大四次会议确定的各项任务，取得了保增长、保民生、保稳定的重大胜利。

--在严峻形势下实现了经济较快增长。初步核算，全年生产总值593.6亿元，增长11.9%;规模以上工业增加值330.3亿元，增长15.1%;城镇固定资产投资284亿元，增长32.7%;地方财政一般预算收入20.4亿元，增长6.3%;社会消费品零售总额186亿元，增长18.5%;城镇居民人均可支配收入13390元，农民人均纯收入5622元，分别增长9.2%和7.1%;出口总额突破1亿美元，增长52.3%。经济增长好于预期，发展态势明显向好。

--在经济低迷状态下实现了招商引资和重点项目建设大突破。全市大招商氛围更加浓厚，县区招商热情空前高涨，第七届中国(漯河)食品博览会和第四届豫商大会成功举办，全年新上亿元以上项目90个，完成投资120亿元，市定重点项目进展顺利，华电2×30万千瓦热电一号机组并网发电，双汇5000万只肉鸡养殖加工、永银盐化工、中粮二期面粉加工等一批重点项目开工建设。08年是我市近年来招商成效最好、开工项目最多、投资力度最大的一年。

--在大灾之年实现了农业生产丰收。面对60年一遇的冬春连旱和秋季飓风等自然灾害，果断采取抗灾措施，积极开展生产自救，全国小麦抗旱春管暨春耕备耕现场会在我市召开，全市粮食总产166.4万吨，夏粮总产、单产连续4年创历史新高。

--在资金困难情况下实现了城乡面貌新变化。多渠道融通建设资金，大力度推进沙澧河开发和城乡基础设施建设，创建国家森林城市通过检查验收，我市成为"中国优秀旅游城市"，城市形象明显提升，综合效应正在彰显，人民群众享受到城乡建设新成果。

--在各种矛盾增多情况下实现了社会和谐稳定。社会事业健康发展，人民生活持续改善，安全生产形势稳定，平安建设成效显著，我市荣获"全国社会治安综合治理优秀市"称号。

折叠工作重点

我们主要抓了以下重点工作:

(一)全力以赴战危机、保增长。面对严峻形势，坚持把保增长作为首要任务，紧紧围绕抓投入、扩内需、拓市场，果敢应对，危中寻机，创造性地开展工作。针对不同阶段的突出矛盾和特点，加强经济预测，及时研究和解决经济运行中遇到的新情况、新问题，扎实开展"企业服务年"活动，通过决战二季度、大干三季度、决胜四季度，全市经济持续回升，在艰难开局情况下实现了良好结局。抢抓政策机遇，争取上级政策性资金6.1亿元，有41个项目纳入中央投资计划。深入实施"重点工业超千亿计划"，97家重点企业完成销售收入935.6亿元。食品、造纸、盐化工三大产业优势更加明显，完成增加值占全市规模以上工业增加值的69%，双汇集团销售收入突破400亿元，银鸽集团生产能力达到100万吨。强力推进产业集聚区建设，编制完成6个产业集聚区发展规划，完成基础设施投入13.6亿元、项目投资114亿元，新入驻项目153个，产业集聚效应初步显现。以光华电子、三鑫稀土等为代表的高新技术企业较快发展。技术创新和名牌战略取得新成果，新引进高新技术项目10个，新培育省级企业技术中心6家、省优质产品9个，国家肉制品质量监督检验中心正式启用。

(二)坚持不懈打基础、强"三农"。认真落实强农惠农政策，向农民发放各类补贴资金6.8亿元。大力调整农业结构，深入推进农业产业化和"双汇产业化工程"，优质粮食播种面积占粮食总面积的97.1%;无公害蔬菜基地发展到62.8万亩，绿色蔬菜基地达到2万亩;新培育标准化养殖小区(场)152个，年出栏生猪317万头;新培育市级龙头企业47家，农民专业合作社发展到454家;完成造林面积12.6万亩，集体林权制度改革走在全省前列;土地流转面积51.6万亩。切实改善农村生产生活条件，新增新农村建设试点村151个、先进示范村35个，规划建设新型农村社区24个，新建改造农村公路262公里、危桥27座、大中型沼气工程14个、户用沼气池3万座，新解决10.4万人安全饮水问题，新打、修缮机井1.2万眼,改善旱涝保收田12万亩，新建农家店264家。村容村貌整治成效明显，4个乡镇、13个村分别荣获省级环境优美乡镇和省级生态文明村。创建"玉米收获机械化示范市"扎实推进，农机化水平进一步提高，农业综合开发、农作物秸秆禁烧及综合利用工作取得新成效，国家农村信息化综合信息服务试点任务圆满完成。

(三)集中精力抓改革、促开放。不断深化企业改革，33家企业实现了战略重组，银鸽集团成功定向增发。金融、财税等改革成效明显，金融机构新增贷款41.2亿元、增长20.4%，剥离不良资产4.2亿元，市商业银行挂牌成立，市城投发债获得批准。政府机构改革稳步推进，高效行政服务机制不断完善。强力实施招商引资百日会战，深入开展以"十年招商大回访"、"境内外豫商大对接"、"产业集聚区大推介"、"行业百强总部大走访"为主题的大招商活动，全市共签约项目141个、已开工项目109个，全年实际利用外资2.5亿美元，提前2个月完成省定目标。

(四)齐心协力建名城、塑形象。不断加大沙澧河开发建设力度，澧河一期建成开园，沙河一期接近尾声，太行山路澧河桥主体工程完工、沙河桥进展顺利，沙河节制闸建成投用，碧水穿城景观已经显现。进一步完善城市功能，对黄河东路、嵩山路等6条道路进行了升级改造，实施了一批道路排水工程，完成市区21条背街小巷改造任务;18个城中村改造试点全面启动;沙北污水处理厂一期工程建成投用;新铺供水管网14公里、燃气管网43公里、热力管网17公里，新建星级公厕20座。强力实施城市环境综合整治，城市综合管理水平明显提高。临颍、舞阳两个县城产业集聚水平明显提升，产城一体步伐加快，小城镇和中心村承载力不断增强，全市城镇化率达到39.3%，城乡一体化建设迈出坚实步伐。

(五)持之以恒办实事、惠民生。各级财政投入22.9亿元用于社会事业建设，省定十大实事、市定十件实事圆满完成。全面落实农村中小学生"两免一补"和城市中小学生免除学杂费政策，受益学生34万人。漯河职院成为省级示范性高等职业院校，漯河医专新校区开工建设，漯河食品职业学院申办成功。公共卫生服务体系进一步完善，新型农村合作医疗参合率达96.7%，建成标准化村卫生室437个、城市社区卫生服务中心10所，改造乡镇卫生院11所，市中医院晋升城市"三甲"，甲型H1N1流感疫情防控工作有力有效。贾湖遗址和许慎墓保护规划通过国家级审批，陈星聚纪念馆建成开馆，许慎文化园、小商桥和杨再兴陵园等重点项目进展顺利。成功举办了中国国际女排精英赛、国际旅游小姐冠军总决赛等重大赛事，提升了漯河的知名度和影响力。群众性体育活动蓬勃开展，"中国排球城"建设正式启动。扎实做好就业再就业工作，新增城镇就业6.5万人，城镇登记失业率3.55%，我市被评为"中国创业之城"，成为河南省创业型城市试点。加快完善社会保障体系，新型农村社会养老保险试点工作正式启动，农村五保集中供养率达67%、居全省第一位，完成了城乡低保提标扩面工作，城镇居民基本医疗保险全面推行，城镇企业离退休人员养老金按时足额发放。新建经济适用房25万平方米，新发放住房公积金贷款2.3亿元。计划生育、环境保护和土地管理三项基本国策得到较好落实，节能减排取得扎实成效。对口援建四川灾区工作走在全省前列。丹江口库区移民安置在全省率先实现一次性整体搬迁。妇女、未成年人、老年人和残疾人合法权益得到切实保障。审计、统计、人防、气象、广播影视、新闻出版、史志档案、民族宗教、外事侨务、慈善事业等都取得新成绩。

(六)千方百计保稳定、促和谐。认真开展学习实践科学发展观活动，持续推进"两转两提"和服务型政府建设。824件人大代表建议和政协委员提案全部办理完毕。精神文明建设得到加强，我市成为全国未成年人思想道德建设先进市、省级文明城市。法治城市建设成效明显，法律援助服务工作走在全省前列。全面加强信访稳定、社会治安、安全生产、应急管理、食品药品监管等工作，我市成为全省平安建设先进市、信访工作先进市、安全生产优秀市、食品安全优秀市。驻漯解放军、武警消防官兵和民兵预备役人员在支持地方建设、维护社会稳定等方面作出了积极贡献，我市连续4次成为全省双拥模范城市。

各位代表!过去的一年，形势复杂，挑战严峻，全市上下和衷共济，攻坚破难，取得的成绩极为不易，这是市委正确领导的结果，是市人大、市政协监督支持的结果，是全市人民团结拼搏的结果。回顾一年的工作，我们深深感到，每一项重点工作的扎实推进、每一阶段经济运行的科学调度、每一次重要活动的成功举办、每一个重大项目的开工建设，都凝结着全市人民的心血和汗水。在此，我代表市人民政府，向全市人民、各民主党派、各人民团体，向驻漯解放军、武警消防官兵、公安民警以及各界人士，向所有关心支持漯河发展的各界朋友，表示衷心的感谢!

在肯定成绩的同时，我们也清醒地认识到，政府工作还没有达到更好的效果，要想使漯河不断地跨越发展和可持续发展，就必须在破解难题上下工夫，在克服不足上求突破。当前，我市经济社会发展中存在的困难和工作中的不足，主要表现为"三难、三低、三不够"。"三难":一是经济转型升级难。我市是以传统产业为主的城市，主导产业特色突出，但产业结构相对单一，经济总量偏小，发展方式还比较粗放，企业自主创新能力不强，内生动力不足。二是中小企业和广大农村融资难。我市尚处在依靠投资拉动阶段，但我市现有存贷款规模小、金融机构力量薄弱，乡镇银行还未设立，企业和农民贷款担保能力差，严重影响着企业的规模膨胀和技术创新，严重影响着全民创业。三是各级财政增收难。税源基础薄弱，新的税收增长点不多，后续财源尚需培育，加之扩大公共服务、全面改善民生的政策性增支因素增多，财政支出压力加大，很多想办该办的事情，还没有办或者没有办好，与广大群众期望仍有较大差距。"三低":一是经济外向度低。出口企业不多，规模不大，产品竞争力不强，对经济的拉动作用有限。二是服务业水平低。生产性服务业发展滞后，与工业发展不相适应，生活性服务业层次较低，消费市场不够活跃，服务业在三次产业中占比不高，仍然是全市经济的"短腿"。三是城镇化水平低。中心城区的人气商气需要加快集聚，县城和小城镇规模偏小，辐射带动能力亟待提高。"三不够":一是发展环境优化不够。一些部门和工作人员发展意识、大局意识、服务意识不够强，不作为、慢作为和乱作为现象时有发生;在建设环境中，还存在着强买强卖、强装强卸、阻工闹事等问题。二是一些干部破解难题的能力不够。对重大问题和重点工作思考不深、研究不透、把握不准，缺乏战术性突破的真招实招，甚至还导致一些失误。三是一些政府部门抓落实的硬度不够。作风不实，落实不力，致使一些重点工作推进不够理想，很多方面存在着一般化现象。

针对这些困难和问题，我们必须高度重视。古人尚能一日三省，我们更应进行反思。一个人需要反思，一座城市更要反思。唯有反思，方知不足;唯有反思，方能超越。我们要以对人民高度负责的使命感和责任感，切实提高各级政府的执行力，牢牢把握工作的主动权，努力把各类问题解决好，把各种矛盾化解好。只有这样，漯河的事情才能办得更好，群众才会更加满意。

折叠编辑本段2010年工作安排

2010年，是决胜"十一五"、谋划"十二五"的关键之年。做好政府工作，任务艰巨，意义重大。当前，全球经济逐渐复苏，国内经济回升向好，国家继续实施积极的 财政政策和适度宽松的货币政策，促进中部地区崛起规划全面实施，产业转移趋势更加明显。我市与沿海地区的政策、体制差异在缩小，区位和市场等优势在扩大，特别是经过能量积聚，基础支撑条件不断完善，形成了较好的发展态势。发展环境总体上会好许多，但是面临的形势也极为复杂，世界经济复苏的基础仍然脆弱，外部环境不稳定、不确定因素依然很多。我们必须正确判断形势，充分利用有利条件和积极因素，周密做好应对各种风险和挑战的准备，勇于迎难而上，敢于排难奋进，善于克难制胜。

政府工作的总体要求是:坚持重在持续、重在提升、重在统筹、重在为民，按照市委整体工作部署，努力在继续解放思想上迈出新步伐，在坚持改革开放上实现新突破，在推动科学发展上取得新进展，在促进社会和谐上见到新成效。

2010年经济社会发展的主要预期目标是:生产总值增长12%，规模以上工业增加值增长16%，地方财政一般预算收入增长10%，全社会固定资产投资增长30%，社会消费品零售总额增长16%，城镇居民人均可支配收入实际增长8%，农民人均纯收入实际增长8%，出口总额增长12%，实际利用外商直接投资增长15%，城镇登记失业率、人口自然增长率、节能减排等指标控制在省定目标以内。

围绕上述目标和要求，要坚定不移地抓好六项重点工作:

(一)坚定不移推进招商引资和重点项目建设。强化开放引领，促进投资拉动，加快新上大项目，倾力培植大企业，推动主导产业扩张升级。重点围绕食品、造纸、化工、纺织、物流等产业，持续深入开展一系列招商引资活动。进一步搭建好招商平台，高水平举办第八届中国(漯河)食品博览会，确保年内签约项目投资总额达到300亿元，新签约超亿元项目60个以上、超5亿元项目6个以上。落实招商责任，实施严格考核，坚决兑现奖惩。优化出口产品结构，在继续抓好肉类、蔬菜、纸品等传统产品出口的同时，努力培育新的出口增长点。实施"124"投资计划，全市安排重点项目100个，完成工业投入200亿元以上，拉动全社会固定资产投资完成400亿元以上。加快重点项目建设，着力抓好双汇5000万只肉鸡养殖加工、银鸽集团10万吨文化纸和18万吨生活纸、永银盐化工、金大地二期、太古可口可乐、喜来登和恒辉两个国际旅游酒店等项目建设。加快推进华电二期项目前期工作，配套抓好天阳热力管网建设和入户工程。抓好石武客运专线建设、京港澳高速公路漯河段拓宽改造和漯阜铁路电气化改造。完善项目建设推进机制，实行目标化管理，促进新上项目早开工、在建项目早投产、投产项目早见效。加强和改进投资管理，严格执行用地、节能、环保、安全等市场准入标准和产业政策，提高项目投资强度。

(二)坚定不移发展壮大工业经济。按照"提升一产、主攻二产、扩大三产"的指导思想，始终坚持工业强市战略不动摇，全面提升工业经济实力。加快建设产业集聚区。以"完善规划、创新机制、构建平台、健全功能、落实政策"为重点，大力实施投资倍增计划和大招商行动计划，强化目标考核，实行奖惩激励，每个产业集聚区确保完成基础设施投资2亿元以上，6个产业集聚区企业营业收入力争突破800亿元。启动漯河新区规划，力争市经济开发区升格为国家级开发区。不断膨胀主导产业。对食品、造纸、盐化工产业，通过政府支持、政策引领，促使企业引进项目、战略重组、技术改造、产品研发，巩固食品产业的龙头地位，壮大造纸工业影响力，打造全省重要的盐化工基地，使三大主导产业的销售收入突破1000亿元，占全部工业的比重达到70%以上。壮大骨干企业群体。实施工业"双百行动计划"，筛选100家重点企业和100家发展快、前景好的高成长性中小企业进行培育。通过三年的努力，使200家企业年销售收入达到1800亿元，力争突破2000亿元。扎实开展服务企业活动。加快中小企业公共服务平台、信息服务网络和小企业创业基地建设，坚决清理和取消不合理收费，对部分小型微利企业实行所得税优惠政策，继续落实财政对中小企业支持政策，培训企业家队伍，推动企业服务工作常态化。加快企业技术创新和新兴产业发展。充分发挥市经济开发区和各产业集聚区的载体作用，抓好电动汽车动力系统、电子产品、生物医药、新材料等一批高新技术产业项目，形成新的增长点。深入实施质量兴市战略，全市省级以上企业技术研发中心达到30家以上，争创全省创新型城市试点。

(三)坚定不移加快新农村建设。继续把解决好 "三农"问题作为全部工作的重中之重，以发展现代农业、增加农民收入、促进农村发展为重点，扎实做好"三农"工作。加快农业结构调整。围绕粮食生产核心区建设，实施粮食产量、品质"双提升行动"，力争粮食生产再夺丰收。大力推进传统农业向现代农业转变，市级现代农业示范园区达到10个、县(区)级50个。扩大无公害、绿色和有机蔬菜规模，全市蔬菜种植面积达到88万亩。加快生态林业建设，完成林业生态工程建设6.4万亩。深入推进农业产业化经营，新培育年销售收入超千万元的龙头企业5个以上。全面实施现代畜牧业发展规划，认真落实财政对畜牧业发展的扶持政策，继续推进"双汇产业化工程"，新培育一批标准化规模养殖小区。大力推进农业标准化，进一步巩固国家级农业综合标准化示范市成果。多渠道增加农民收入。全面落实强农惠农政策，继续实施好种粮直补、农资综合直补、良种补贴和农机具购置补贴，认真落实家电、汽车、摩托车下乡和汽车以旧换新等政策。改善农民工就业服务，强化技能培训，新增转移就业农村劳动力3万人以上。

大力发展劳务经济，组织农民工到发达地区就业，引导农民工在当地从业，扶持农民工回乡创业，最大限度地拓展农民增收空间。加强农村基础设施建设。坚持财政支出优先支持农业农村发展，预算内固定资产投资优先投向农业基础设施和农村民生工程，土地出让收益优先用于农业土地开发和农村基础设施建设。深入实施"两县十镇百村示范带动工程"，新建35个综合水平较高的示范村。新建改造农村公路150公里，改造危桥10座，发展农村沼气用户1万户，新解决6万人以上的安全饮水问题，完成215个村的村容村貌整治任务，行政村"农家店"覆盖率达到100%，农业信息覆盖农户70%以上，积极创建"玉米收获机械化示范市"，综合机械化水平进入全省先进行列。深化生态文明村镇创建工作，建成10个省级生态文明村、3个省级环境优美小城镇。深化农村各项改革。创新农村投融资机制，解决农民贷款抵押难、担保难问题。大力推进农村土地流转，积极探索股份合作、企业租赁等多种流转模式。深化农村信用社改革，积极发展村镇银行、小额贷款公司。加快推进城乡一体化。坚持产城一体，扩大临颍、舞阳城区规模。完成乡镇总体建设规划，加快编制中心村规划。按照"因地制宜、统筹规划、分步实施"的原则，加大对农村新型社区的支持力度。以近郊"十镇"和市定重点镇为突破口，全力推进小城镇建设，着力解决道路硬化、供排水、沼气、污水处理等方面的问题，逐步建立组保洁、村收集、乡运输、县处理的农村生活垃圾处理新机制。积极推进户籍制度改革，进一步放宽城镇落户条件。有计划、有步骤解决好农民工在城镇的就业和生活问题，逐步实现农民工在劳动报酬、子女就学、公共卫生、住房租购及社会保障方面与城镇居民享有同等待遇。大力发展县区经济，支持县区做大做强主导产业，培育乡镇特色经济，完善目标管理体系，激励县区争先晋位，促进县区经济跨越发展。

(四)坚定不移提升城市带动功能。当前，城市发展已由产业带动城市转变为城市拉动产业，城市已经成为区域经济发展的龙头。我们要瞄准百万人口城市的目标，不断拉大城市框架，科学优化城市布局，提升城市宜居品质，实现城市功能的最大化，增强城市对经济的带动能力。科学编制城市规划。高标准修编城市总体规划，加快编制各类专业规划和控制性详细规划，依法维护规划的权威性和严肃性。强力推进沙澧河开发建设。高质量完成沙河一期，"五一"前向市民开放，开工建设沙河二期(金山路桥-淞江路桥段)，启动沙澧河交汇处重点区域开发项目，加快沙澧河旅游项目建设及凤凰岛开发步伐，把漯河打造成滨水特色更加凸显的城市，努力彰显碧水穿城、林茂花艳、建筑精巧、环境秀美的城市风貌，致力打造美丽漯河。不断完善城市基础设施。加快推进老城区和太行山路、中山路等6条市区主干道升级改造，建成太行山路沙河桥、澧河桥，启动解放路沙河大桥设计工作。双汇广场建成投用。开工建设城市规划展示馆。加快推进西城区客运站及配套设施建设。新改造15条背街小巷和一批城中村，新增城市供水管网15公里、燃气管网15公里、热力管网12公里，建成东城污水处理厂，推进市垃圾填埋场二期等工程建设，新建改造公厕15座、垃圾中转楼5座。推进景观照明、城市园林雪灾后绿化恢复和金山路、人民路绿化改造等工程，城市空气质量优良天数达330天以上，澧河饮用水源水质达标率保持在100%。切实提高城市管理水平。

深入推进"五城同创"，实施市容市貌提升计划，完善城市管理体制机制，提高城市精细化、网格化、数字化管理水平。加大违法建筑治理力度，突出抓好城市主干道、重要地段、城乡结合部等区域环境整治，逐步建立建筑立面改造更新制度。进一步强化城市产业支撑。突出发展商贸物流业，支持千盛百货、新玛特、丹尼斯等重点市场发展，加快建设中国漯河国际食品城、双河农产品批发市场、福田食品批发交易市场，改造铁东农贸市场;支持冷链物流、盐化工物流、粮食物流、口岸物流等做大做强，推进淞江仓储物流园区建设。大力发展餐饮业，承办高规格烹饪赛事，逐步把漯河建成全国美食名吃集聚地;实施"放心早餐工程"，标准化早餐经营网点达到200个。着力发展房地产业，优化住房结构，加强房地产市场管理，有计划地实施旧住宅小区整治，提高住宅小区物业管理水平。加快发展文化休闲娱乐业，鼓励支持金融、保险、会展、技术服务、文化传媒、信息咨询等产业健康发展。通过不懈努力，力争把城市规模做大、产业做强、环境做美，不断增强漯河的影响力、辐射力和带动力。

(五)坚定不移保障和改善民生。经济发展的根本目的在于改善民生，政府工作的头等大事在于保障民生。要继续加大民生保障和改善力度，让人民群众得到更多实惠。做好就业再就业工作。深入开展全民创业活动，以创业带动就业。广泛开展"万人创业帮扶"活动，做好高校毕业生、城镇转业退伍军人就业工作，为"4050"人员开发更多公益性就业岗位。全年新增城镇就业人员3万人，下岗失业人员再就业1.2万人。完善社会保障体系。扩大养老、医疗、失业、工伤、生育等各项保险覆盖面，把全市关闭破产的国有、集体企业符合条件退休人员全部纳入基本养老保险范围。继续提高企业退休人员养老金标准，幅度不低于全市养老金平均水平的10%。积极推进新型农村社会养老保险试点。完善城乡最低生活保障制度，实现动态管理下应保尽保。加大对低收入家庭补贴和救助力度，做好生活无着落人员的救助工作。切实加强住房保障，开工建设经济适用房15万平方米、廉租房6万平方米，积极推进农村危房改造和城市棚户区改造。进一步加大社会保障投入，加快构建更加完善的社会保障安全网，使人民生活有基本保障、无后顾之忧。加快发展各项社会事业。坚持优先发展教育，进一步优化教育结构，大力推进素质教育，鼓励社会力量兴办教育，认真落实困难家庭学生资助政策，促进城乡教育均衡发展。实施好中小学校舍安全工程，逐步使所有学校的校舍、设备和师资达到规定标准。逐步解决县区教师工资偏低问题。解决好进城务工人员随迁子女平等接受义务教育和留守儿童义务教育问题。支持漯河职院、漯河医专和食品职业学院建设，启动职业教育"双十工程"，选择10所职业学校，经过3年培育，在校生达到10万人。加强公共卫生和医疗服务体系建设，提高新型农村合作医疗保障水平。加大对社区卫生服务投入，保证人均基本公共卫生服务经费标准不低于15元。建成市中医院新门诊楼、市妇幼保健大楼，启动市精神病专科医院建设项目，改造一批县区医院、乡镇卫生院和村卫生室。继续做好甲型H1N1流感等重大疫情防控工作。筹办好2010年中国国际女排精英赛、世界男排联赛等体育赛事，积极备战全省第十一届运动会，加快推进"中国排球城"建设，建成市游泳馆。实施科教兴漯和人才强市战略，吸引更多的高层次、创新型人才来漯创业。繁荣发展文化事业，新建一批乡镇综合文化站和农家书屋。举办第二届许慎文化国际学术研讨会。完成有线电视数字化整体转换和双向改造。按照上级部署，积极推进社会事业改革。加快旅游业开发步伐，高标准推进小商桥和杨再兴陵园等重点项目建设，许慎文化园10月1日前建成开放，加大舞阳农民画、南街村纪念品等特色旅游商品的开发力度，实施旅游富民工程，培育一批新的生态旅游示范点。
